# Gastrointestinal microbiome of ARDS patients induces neuroinflammation and cognitive impairment in mice

**DOI:** 10.1186/s12974-023-02825-7

**Published:** 2023-07-15

**Authors:** Hong Zheng, Qihui Zhao, Jianuo Chen, Jiahui Lu, Yuping Li, Hongchang Gao

**Affiliations:** 1 Oujiang Laboratory, School of Pharmaceutical Sciences, Wenzhou Medical University, Wenzhou, 325035 China; 2 Department of Pulmonary and Critical Care Medicine, The First Affiliated Hospital of Wenzhou Medical University, Wenzhou, 325015 China

**Keywords:** ARDS, Behavior, Gut-brain axis, Inflammation, Microglia

## Abstract

**Background:**

Acute respiratory distress syndrome (ARDS) is a respiratory failure syndrome that can cause many complications, impacting patients’ quality of life. Behavioral and cognitive disorders have attracted increasing attention in patients with ARDS, but its potential mechanisms are still elusive.

**Methods:**

Herein we transferred the faecal microbiota from patients with ARDS caused by community-acquired pneumonia (CAP) to antibiotics-treated recipient male mice to explore the microbiota-gut-brain mechanisms. Behavioral functions of mice were evaluated by the open field test, Morris water maze and Y-maze test. The structure and composition of the gut microbiota were analyzed by using 16S rRNA sequencing analysis. Microglia, astrocyte and neuron in the cortex and hippocampus were examined via immunofluorescent staining.

**Results:**

We found that the major characteristic of the intestinal flora in ARDS/CAP patients was higher abundances of Gram-negative bacteria than normal controls. The gut microbiota derived from ARDS/CAP patients promoted neuroinflammation and behavioral dysfunctions in mice. Mice who underwent fecal transplant from ARDS/CAP patients had increased systemic lipopolysaccharide (LPS), systemic inflammation, and increased colonic barrier permeability. This may adversely impact blood barrier permeability and facilitate microglia activation, astrocyte proliferation, and loss of neurons.

**Conclusions:**

Our study proposes the role of the microbiota-gut-brain crosstalk on ARDS/CAP-associated behavioral impairments and suggests the gut microbiota as a potential target for the protection of brain health in ARDS patients in clinical practice.

**Supplementary Information:**

The online version contains supplementary material available at 10.1186/s12974-023-02825-7.

## Introduction

Acute respiratory distress syndrome (ARDS) as a syndrome of respiratory failure is caused by noncardiogenic pulmonary edema, leading to high mortality and financial cost [1]. ARDS often occurs in patients with pneumonia, sepsis, severe trauma and COVID-19 and even one in ten patients develop into ARDS in intensive care units [2]. Of particular note, ARDS can lead to a series of complications such as depression, anxiety, critical illness myopathy, fibrosis, pulmonary function decline, posttraumatic stress disorder and ambulatory dysfunction [3]. In addition, cognitive impairment and memory loss have been also recognized as ARDS-associated complications, which might be attributed to inflammation, hypoxemia and blood brain barrier disruption [4]. The impact of ARDS on brain health is gaining increasing attention, but the possible pathogenesis still need to be further explored.

The gut microbiota has been associated with the onset and progress of ARDS [5]. Moreover, of note, accumulating evidence suggests that the disturbance of gut microbiota contributes to behavior and brain disorders [6]. For example, Neufeld et al. found that the absence of the intestinal microbiota alleviated anxiety-like behavior in mice [7]. Antibiotic-induced gut dysbiosis resulted in cognitive decline of mice via regulating circulating metabolites and cerebral neuropeptide Y system [8]. In our previous study, we also revealed that depletion of acetate-producing bacteria from the gut microbiota by vancomycin reduced hippocampal synaptophysin level and impaired abilities of learning and memory in mice through the vagus nerve stimulation [9]. In addition to germ-free and antibiotic-treated animal studies, faecal microbiota transplantation (FMT) is a commonly used approach to explore the relationship between the gut microbiota and brain functions. Zheng et al. reported that the gut microbiota in schizophrenia patients can disrupt neurotransmitter metabolism and behaviors in mice [10]. Transfer of the intestinal microbiota from patients with major depressive disorder leaded to altered host metabolism and depression-like behaviors in germ-free mice [11]. Transplantation of the faecal microbiota from healthy wild-type mice alleviated memory impairment via lowering amyloid and tau pathology in ADLP^APT^ mice [12]. FMT from healthy mice improved gastrointestinal dysfunction and the motor deficit in rotenone-induced Parkinson’s disease by suppression of inflammatory response [13]. In addition, the causal connections between the gut microbiota and brain health during aging were also established by FMT between young and aged mice [14, 15]. These studies indicated that the gut microbiota can mediate the gut-brain crosstalk and affect brain functions. Thus we hypothesize that ARDS microbiota may also affect brain health via the gut-brain axis.

In the present study, we analyzed the gut microbial characteristics of ARDS patients caused by community-acquired pneumonia (CAP) and then transferred ARDS/CAP microbiota to antibiotics-treated recipient mice for investigating its influences on brain health. The aims of this study are (1) to examine the effect of ARDS/CAP microbiota on behavior and brain functions and (2) to explore potential gut-brain axis mechanisms. Our results may provide a new perspective to prevent or treat ARDS-associated brain disorders by targeting the gut-brain axis.

## Results

### ARDS/CAP patients exhibit a shift in the gut microbiota composition

In the present study, a total of 21 ARDS/CAP patients and 21 healthy controls (CTRL) were recruited from the affiliated hospital of our university. There were no significant differences in gender and age between ARDS/CAP and CTRL groups (Additional file 1: Table S1). Of note, ARDS/CAP patients had drastically increased levels of procalcitonin, C-reactive protein and IL-6, which are far more than the normal ranges (Additional file 1: Table S1), suggesting a high inflammatory response. Yet, the levels of IL-2, IL-4 and TNF-α in ARDS/CAP patients were still within the normal ranges (Additional file 1: Table S1).

**Fig. 1:**
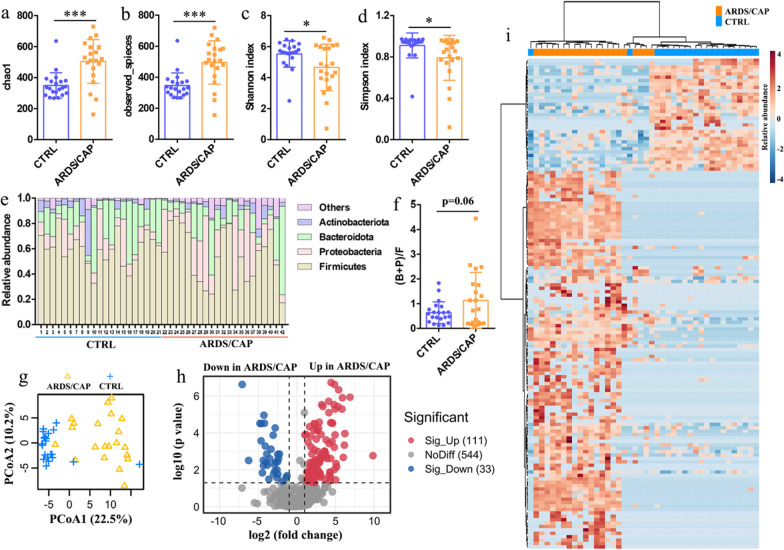
Characteristics of the gut microbiota in ARDS/CAP patients. **a** chao1 index. **b** observed species. **c** Shannon index. **d** Simpson index. **e** The relative abundances of the gut microbiota at the phylum level. **f** The ratio of (Bacteroidota + Proteobacteria)/Firmicutes. **g** PCoA based on the gut microbiota at the genus level. **h** Volcano plot analysis identifying key gut microbes that significantly altered between ARDS/CAP patients and normal control (CTRL) subjects. **i** Heatmap showing the key gut microbes between ARDS/CAP patients and CTRL subjects. The statistic difference of various indicators between two groups was evaluated by two-tailed unpaired student’s T test and a statistically significant was defined when p < 0.05. Significant level: *p < 0.05; ***p < 0.001

Then we examined the characteristics of the gut microbiome in faecal samples of ARDS/CAP patients by 16S rRNA sequencing analysis. The results reveal that ARDS/CAP patients had significantly higher levels of chao1 (Fig. 1a) and observed species (Fig. 1b) but lower Shannon (Fig. 1c) and Simpson (Fig. 1d) indexes relative to CTRL subjects. These findings suggest that total number of species was increased but the diversity of species was decreased in ARDS/CAP patients. Figure 1e shows that the relative abundances of the gut microbiota at the phylum level were altered in ARDS/CAP patients. The ratio of (Bacteroidota + Proteobacteria)/Firmicutes, (B + F)/F, was higher in ARDS/CAP patients than CTRL subjects (p = 0.06, Fig. 1f), indicating that ARDS/CAP patients had an enriched abundance of Gram-negative bacteria. A clear separation between ARDS/CAP and CTRL groups based on the PCoA analysis of the gut microbiota at the genus level suggests that ARDS/CAP patients exhibited a differentiated microbial pattern relative to CTRL subjects (Fig. 1g). Afterward, volcano plot analysis was employed to identify key gut microbes that significantly altered between ARDS/CAP and CTRL groups (Fig. 1h), and presented as a heatmap (Fig. 1i) and listed in Additional file 2. In total of 144 gut microbes were identified, of which 111 (77.08%) gut microbes were significantly increased in ARDS/CAP patients compared with CTRL subjects (Additional file 1: Table S2). We also found that 39 Gram-negative bacteria were enriched in ARDS/CAP patients including 29 (93.55%) in *Proteobacteria* and 10 (83.33%) in *Bacteroidota* (Additional file 1: Table S2). Collectively, our results reveal that ARDS/CAP patients had a significant shift in the gut microbiota especially increased abundances of Gram-negative bacteria.

### Microbiota transfers from ARDS/CAP patients to antibiotics-treated recipient mice

**Fig. 2:**
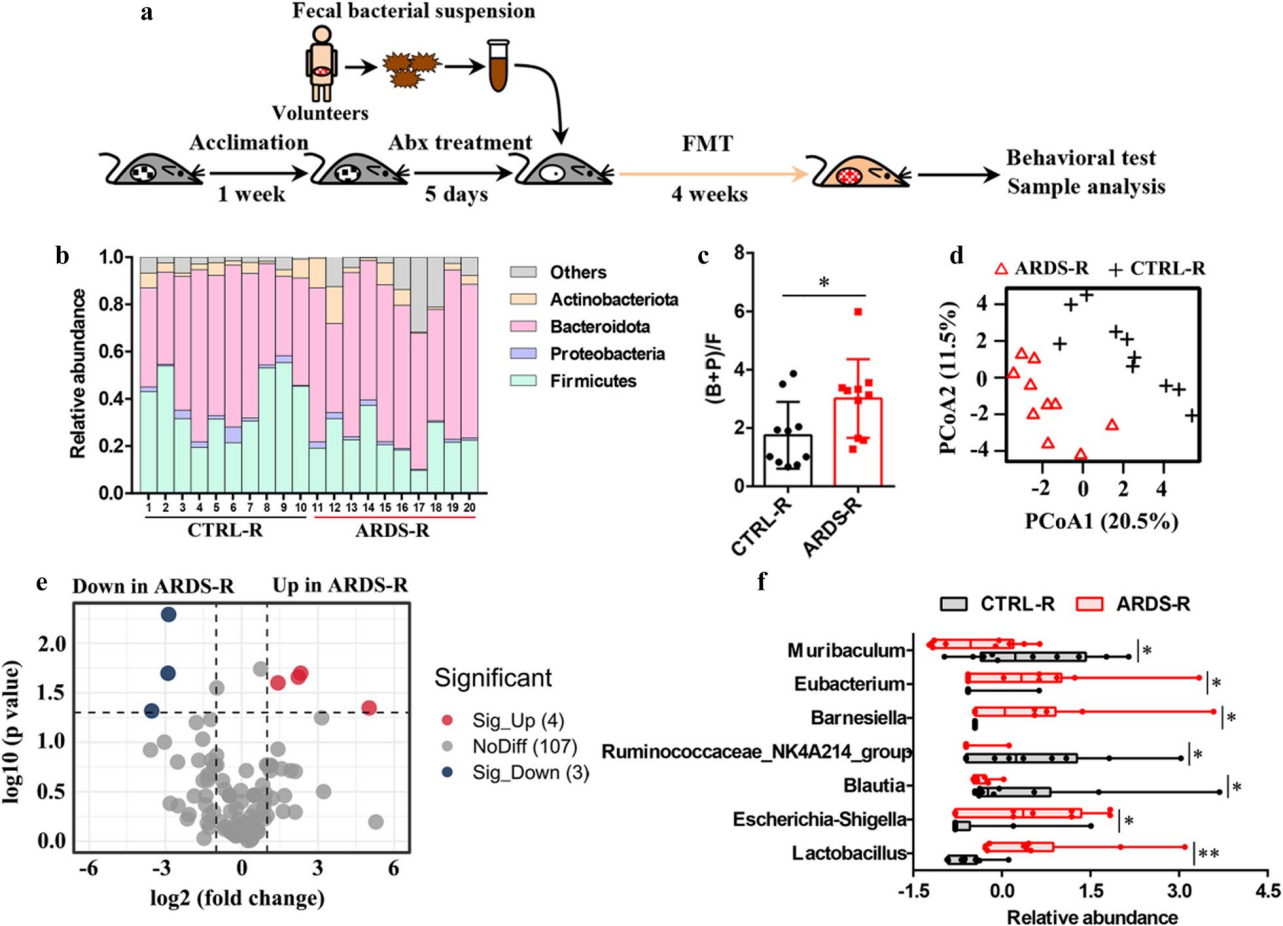
The gut microbiota transfers from ARDS/CAP patients to recipient mice. **a** Flow diagram of FMT experiment: After 1 week of acclimation, mice were treated with an antibiotic (Abx) cocktail for 7 days. Then, faecal material from ARDS/CAP patients or normal control (CTRL) subjects was transferred to Abx-treated recipient mice (ARDS-R and CTRL-R) for 4 weeks. **b** Changes in the relative abundances of the gut microbiota at the phylum level between ARDS-R and CTRL-R mice. **c** Changes in the ratio of (Bacteroidota + Proteobacteria)/Firmicutes between ARDS-R and CTRL-R mice. **d** PCoA based on the gut microbiota at the genus level. **e** Volcano plot analysis identifying key gut microbes that significantly altered between ARDS-R and CTRL-R mice. **f** Changes in the relative abundances of the key gut microbes between ARDS-R and CTRL-R mice. The statistic difference of various indicators between two groups was evaluated by two-tailed unpaired student’s T test and a statistically significant was defined when p < 0.05. Significant level: *p < 0.05

To study the impact of ARDS/CAP microbiota on brain health, the faecal microbiota from either ARDS/CAP patients or CTRL subjects was transferred to antibiotics-treated recipient male mice once a day for 4 weeks, and named as ARDS-R and CTRL-R in this study, respectively (Fig. 2a). Relative to CTRL-R mice, the relative abundances of the gut microbiota at the phylum level were altered in ARDS-R mice (Fig. 2f), where the ratio of (B + F)/F was significantly higher in ARDS-R mice (Fig. 2g), implying that increased Gram-negative bacteria in ARDS/CAP patients was transferred to recipient mice. The PCoA analysis of the gut microbiota at the genus level reveals that ARDS-R mice displayed a clearly discriminative microbial pattern when compared with CTRL-R mice, as illustrated in Fig. 2h. Then significantly altered gut microbes between ARDS-R and CTRL-R mice were identified by volcano plot analysis (Fig. 2i) and presented in Fig. 2j. The results show that ARDS-R mice had significantly higher abundances in *Eubacterium, Barnesiella, Escherichia-Shigella* and *Lactobacillus* but lower *Muribaculum, Ruminococcaceae_NK4A214_group* and *Blautia* relative to CTRL-R mice (Fig. 2j). Thereinto, higher abundances of *Eubacterium, Barnesiella, Escherichia-Shigella* and *Lactobacillus* in ARDS/CAP patients were transferred to recipient mice.

### ARDS/CAP microbiota causes colonic barrier injury and systemic inflammation

**Fig. 3:**
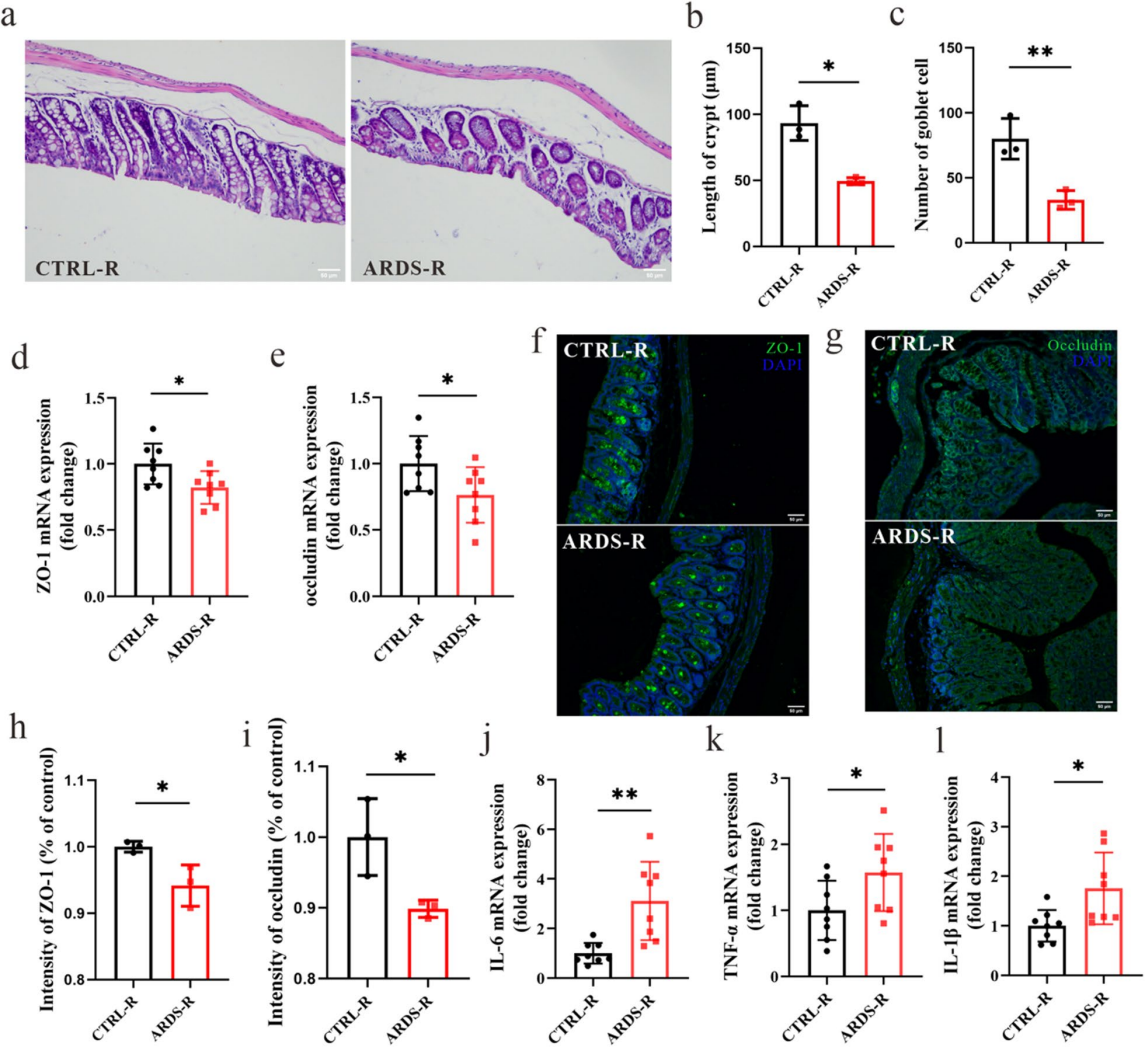
The gut microbiota from ARDS/CAP patients causes colonic barrier injury and inflammation in mice. **a** Hematoxylin/Eosin (HE) staining showing the colonic structure changes in mice receiving the faecal microbiota from ARDS/CAP patients (ARDS-R) or normal control subjects (CTRL-R); Image scale: 200 × magnification. (**b**) Length of colonic crypt. **c** The number of colonic goblet cells. **d, e** The mRNA expression levels of (**d**) ZO-1 and (**e**) Occludin in the colon of ARDS-R and CTRL-R mice. **f, h** Immunofluorescent assay of colonic ZO-1 and the corresponding quantitative result; Image scale: 200 × magnification. **g, i** Immunofluorescent assay of colonic Occludin and the corresponding quantitative result; Image scale: 200 × magnification. **j–l** The mRNA expression levels of (**j**) IL-6, (**k**) TNF-α and (**l**) IL-1β in the colon of ARDS-R and CTRL-R mice. The statistic difference of various indicators between two groups was evaluated by two-tailed unpaired student’s T test and a statistically significant was defined when p < 0.05. Significant level: *p < 0.05; **p < 0.01

To investigate the impact of ARDS/CAP microbiota on the intestinal tract, we analyzed the morphology, inflammation and tight junction proteins in the colon of mice. We found that the length of crypt (Figs. 3a and b) and the number of goblet cells (Fig. 3a and c) were significantly reduced in ARDS-R mice compared with CTRL-R mice, indicating a disrupted intestinal homeostasis in mice receiving ARDS/CAP microbiota. Relative to CTRL-R mice, the mRNA expression levels of ZO-1 (Fig. 3h) and occludin (Fig. 3i) were significantly lower in the colon of ARDS-R mice. Moreover, immunofluorescent assay confirmed that ARDS-R mice had significantly reduced ZO-1 (Fig. 3d and e) and occludin (Figs. 3f and g) at the protein level than CTRL-R mice. These results reveal that colonic barrier integrity was damaged in mice receiving ARDS/CAP microbiota. In addition, ARDS/CAP microbiota may also promote colonic inflammatory response in mice as indicated by significantly increased levels of IL-6, TNF-α and IL-1β (Fig. 3j–l).

Serum lipopolysaccharide (LPS) was significantly increased in ARDS-R mice compared to CTRL-R mice which may be due to increased intestinal barrier permeability (Additional file 1: Figure S1a). This finding is in agreement with the clinical results in ARDS/CAP patients as indicated by significantly increased levels of intestinal permeability indicators including DAO (Diamine oxidase, p = 0.001) and LPS (p = 0.015) (Additional file 1: Table S1). LPS has been recognized as a key pro-inflammatory product derived from the gut microbiota, resulting in inflammatory infiltrate and M1 macrophage activation in the lung of ARDS-R mice observed from HE staining and F4/80 immunostaining, respectively (Additional file 1: Figure S1b). Moreover, we also detected significantly higher levels of IL-6 (Additional file 1: Figure S1c) and TNF-α (Additional file 1: Figure S1d) in the lung of ARDS-R mice relative to CTRL-R mice. The ELISA assay shows that ARDS-R mice had drastically increased IL-6 (Additional file 1: Figure S1e) and TNF-α (Additional file 1: Figure S1f) in the serum than CTRL-R mice. Taken together, our results suggest that the gut microbiota from ARDS/CAP patients may promote inflammatory response by destroying colonic barrier integrity and increasing endotoxin influx.

### ARDS/CAP microbiota promotes microglia activation, neuroinflammation and neuron loss in mice

**Fig. 4:**
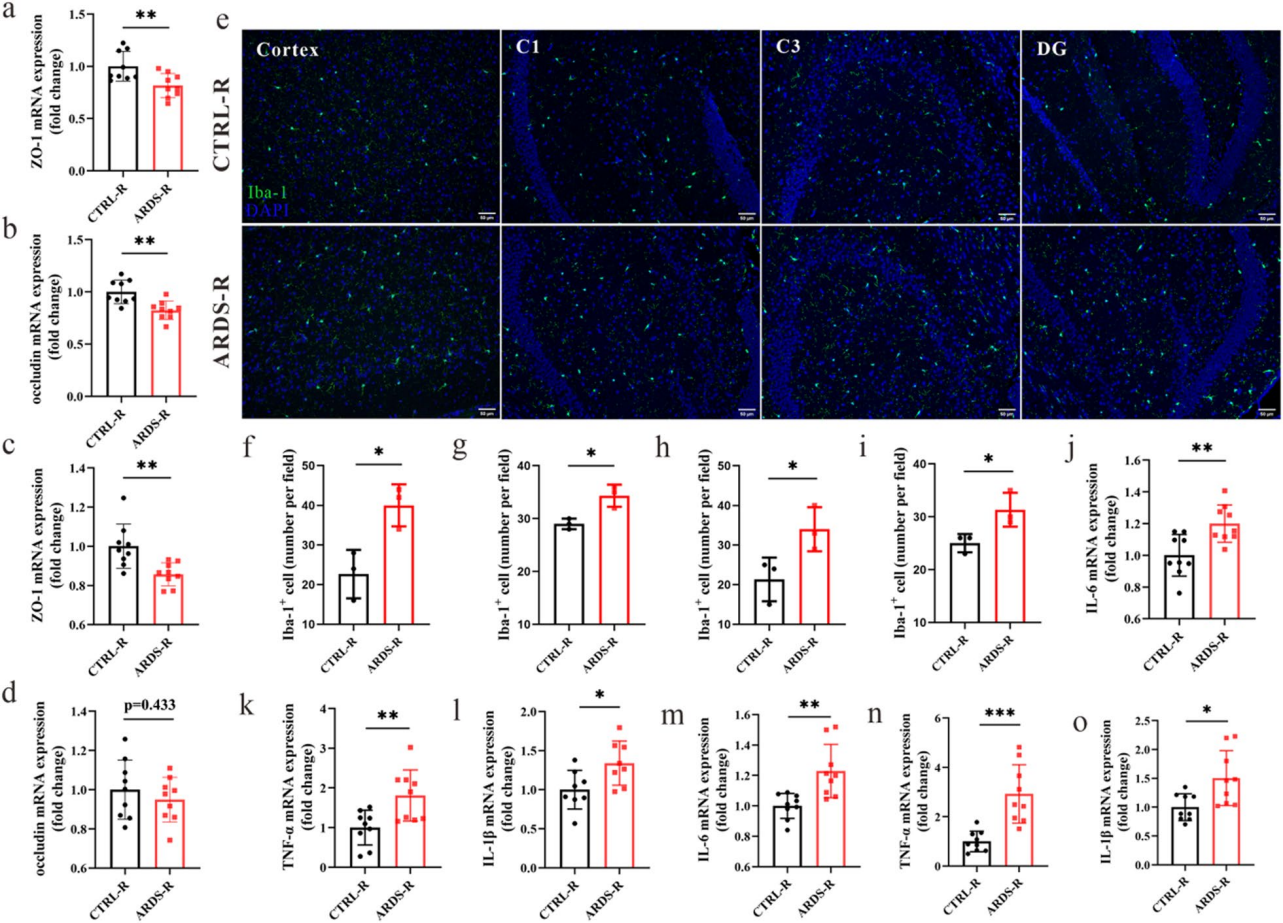
The gut microbiota from ARDS/CAP patients promotes microglia activation and neuroinflammation in the brain of mice. **a, b** The mRNA expression levels of (**a**) ZO-1 and (**b**) occludin in the cortex of mice receiving the faecal microbiota from ARDS/CAP patients (ARDS-R) or normal control subjects (CTRL-R). **c, d** The mRNA expression levels of (**c**) ZO-1 and (**d**) occludin in the hippocampus of ARDS-R and CTRL-R mice. **e** Typical Iba-1 immunostaining showing the density of activated microglia in the cortex and different hippocampal regions including C1, C3 and DG; Image scale: 200 × magnification. **f–i** The corresponding quantitative results of Iba-1 positive cells in the (**f**) cortex and hippocampal (**g**) C1, (**h**) C3 and (**i**) DG regions. **j–l** The mRNA expression levels of (**j**) IL-6, (**k**) TNF-α and (**l**) IL-1β in the cortex of ARDS-R and CTRL-R mice. **m–o** The mRNA expression levels of (**m**) IL-6, (**n**) TNF-α and (**o**) IL-1β in the hippocampus of ARDS-R and CTRL-R mice. The statistic difference of various indicators between two groups was evaluated by two-tailed unpaired student’s T test and a statistically significant was defined when p < 0.05. Significant level: *p < 0.05; **p < 0.01; ***p < 0.001

In this study, we found that the mRNA expression levels of ZO-1 (Fig. 4a) and occludin (Fig. 4a) were significantly reduced in the cortex of ARDS-R mice relative to CTRL-R mice. In the hippocampus, ARDS-R mice exhibited a lower ZO-1 level than CTRL-R mice (Fig. 4c), whereas no significant difference was obtained in the occludin level between two groups (Fig. 4d). These results imply that ARDS/CAP microbiota may disrupt blood brain barrier integrity. Then we used Iba-1 staining to examine the density of activated microglia in the cortex and hippocampus of ARDS-R mice, and representative Iba-1 staining were illustrated in Fig. 4e. The quantitative results show that the number of Iba-1 positive cells was significantly increased in the cortex (Fig. 4f) and hippocampus including C1 (Fig. 4g), C3 (Fig. 4h) and DG (Fig. 4i) regions of ARDS-R mice relative to CTRL-R mice. Additionally, the mRNA expression levels of the pro-inflammatory cytokines released from activated microglia including IL-1β, IL-6 and TNF-α were also drastically higher in the cortex (Fig. 4j–l) and hippocampus (Fig. 4m–o) of ARDS-R mice than CTRL-R mice. The number of astrocyte and neuron in the cortex and hippocampus were examined by GFAP and NeuN immunofluorescent staining and representative images were illustrated in Additional file 1: Figures S2a and S2f, respectively. The corresponding quantitative results reveal that the number of GFAP positive cells was significantly increased in the cortex (Additional file 1: Figure S2b) and hippocampus including C1 (Figure S2c), C3 (Additional file 1: Figure S2d) and DG (Additional file 1: Figure S2e) regions of ARDS-R mice when compared with CTRL-R mice. Yet, ARDS-R mice exhibited significant reductions in the number of NeuN positive cells in the cortex (Additional file 1: Figure S2g) and hippocampal C1 (Additional file 1: Figure S2h), C3 (Additional file 1: Figure S2i) and DG (Additional file 1: Figure S2j) than CTRL-R mice. Collectively, ARDS/CAP microbiota may cause microglia activation and astrocyte proliferation in mice, leading to neuroinflammation and neuron loss.

### The gut microbiota from ARDS/CAP patients induces behavioral disorders in mice

**Fig. 5:**
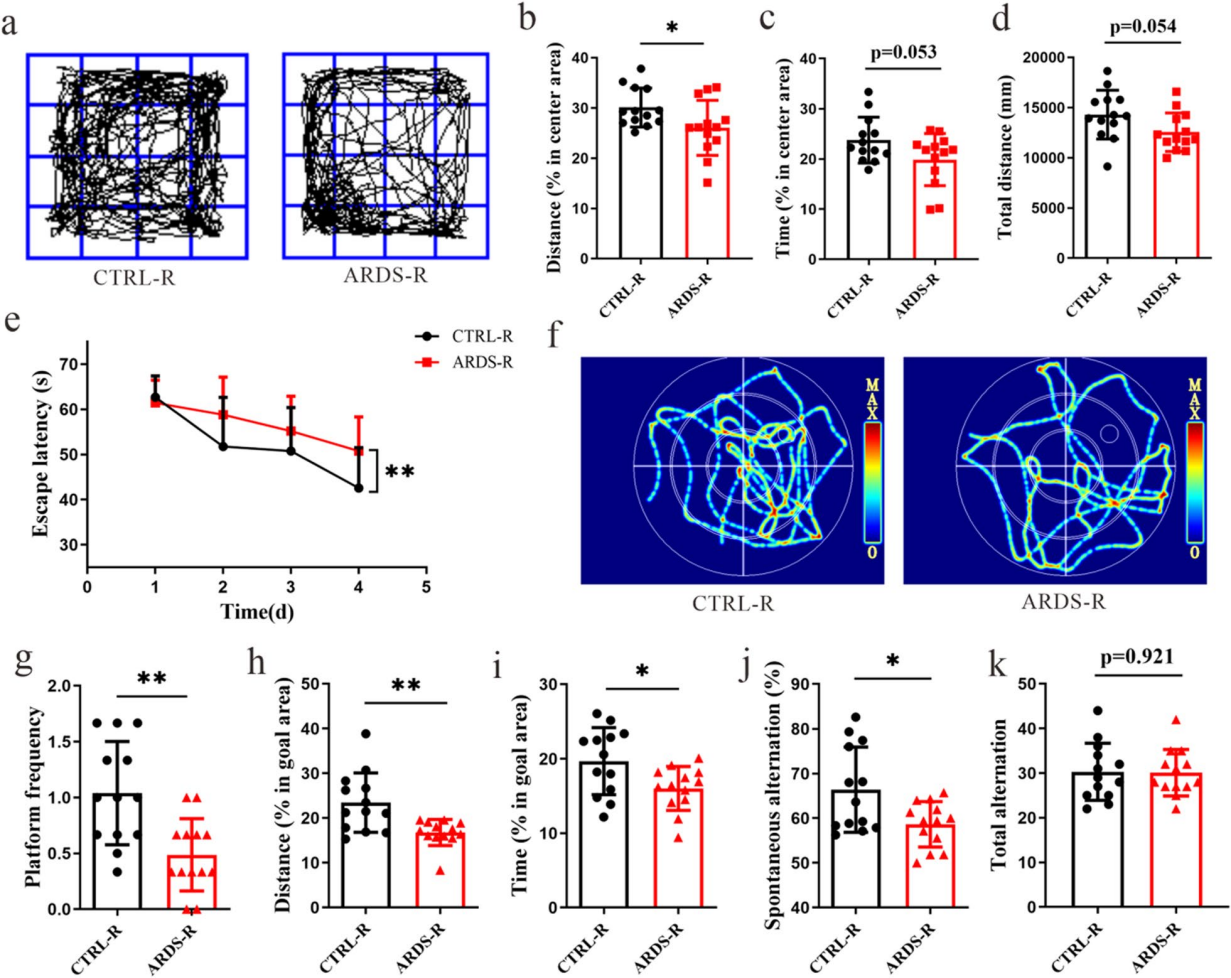
The gut microbiota from ARDS/CAP patients induces behavioral disorders in mice. **a** Typical movement trajectories of mice receiving the faecal microbiota from ARDS/CAP patients (ARDS-R) or normal control subjects (CTRL-R) in the open field test (OFT). **b–d** The percentages of (**b**) distance and (**c**) time in the central area and (**d**) total distance in the OFT. **e** The escape latency of ARDS-R and CTRL-R mice during training in the Morris water maze (MWM). **f** Typical swimming trajectories of CTRL-R and ARDS-R mice during testing in the MWM. **g–i** The platform frequency (**g**) and the percentage of distance (**h**) and time (**i**) in goal area of CTRL-R and ARDS-R mice during testing in the MWM. **j, k** The spontaneous alternation (**j**) and total alternation of CTRL-R and ARDS-R mice in the Y-maze test. The statistic difference in the change of the escape latency between two groups was analyzed via a repeated measure ANOVA. The statistic difference of various indicators between two groups was evaluated by two-tailed unpaired student’s T test and a statistically significant was defined when p < 0.05. Significant level: *p < 0.05; **p < 0.01

To assess the effect of ARDS/CAP microbiota on behavioral functions of mice, open field test (OFT), Morris water maze (MWM) and Y-maze test (YMT) were carried out in this study. Figure 5a illustrates the typical movement trajectories of CTRL-R and ARDS-R mice during the OFT, where we can see that ARDS-R mice had lower percentages of distance (Fig. 5b, p < 0.05) and time (Fig. 5c, p = 0.053) in the central area and total distance (Fig. 5d, p = 0.054). Therefore, the OFT results reveal that anxiety-like behaviors appeared in ARDS-R mice. The MWM results show that the escape latency during training was significantly longer in ARDS-R mice than CTRL-R mice (Fig. 5e), suggesting that learning ability was impaired in ARDS-R mice. Subsequently, the escape platform was removed to test memory ability at day 5 after training and the typical swimming trajectories of CTRL-R and ARDS-R mice were illustrated in Fig. 5f. We found that ARDS-R mice had an impaired memory ability as characterized by significantly reductions in platform frequency (Fig. 5g) and the percentage of distance (Fig. 5h) and time (Fig. 5i) in goal area relative to CTRL-R mice. Additionally, compared with CTRL-R mice, spontaneous alternation in the YMT was significantly reduced in ARDS-R mice (Fig. 5j), whereas no significant change was observed in total alternation (Fig. 5k). This finding indicates that spatial working and reference memory were damaged in ARDS-R mice. Hence, our results suggest that ARDS/CAP microbiota can result in behavioral disorders in mice.

### Behavioral disorders are associated with the gut microbiota, barrier function and inflammation

**Fig. 6:**
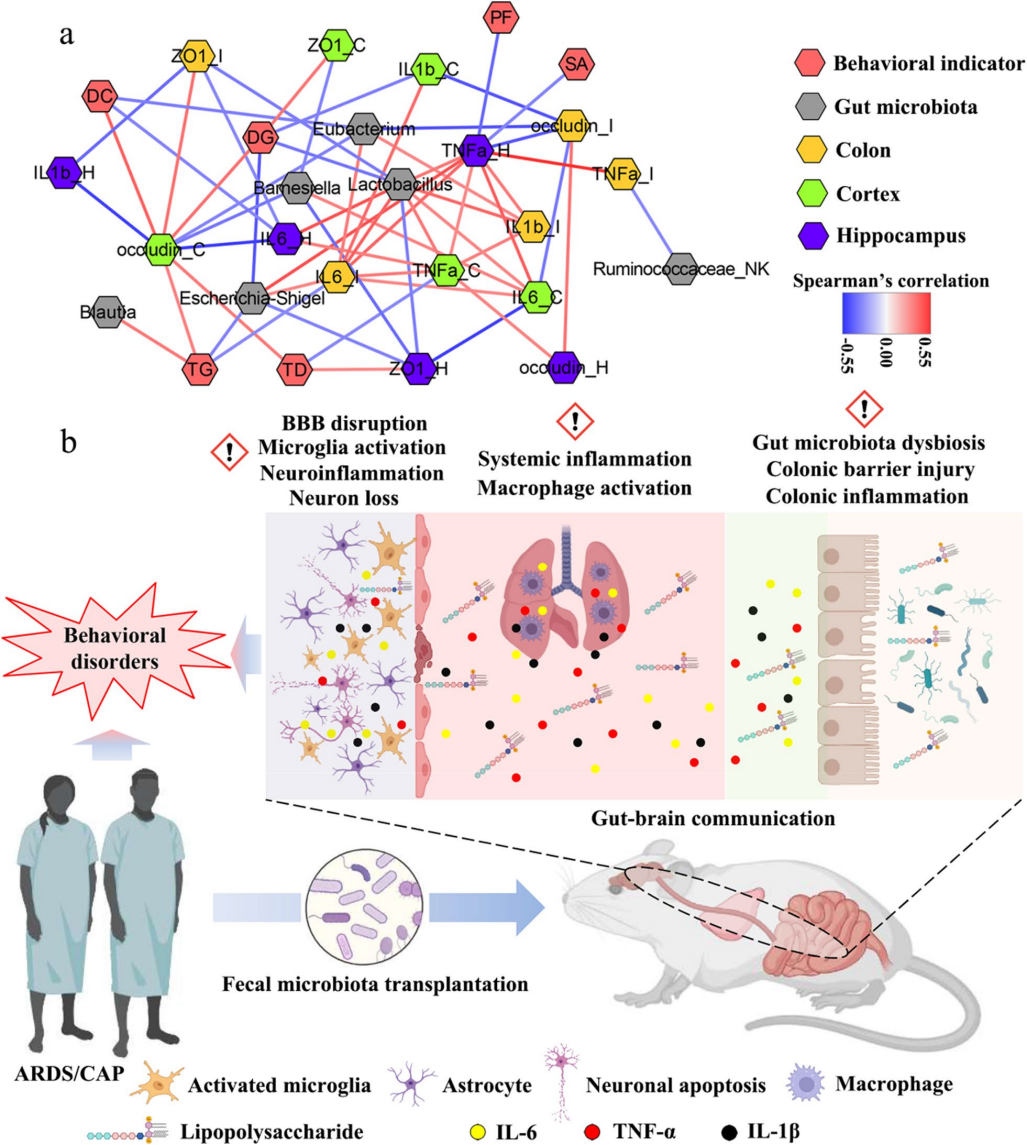
The microbiota-gut-brain crosstalk in ARDS/CAP patients. **a** Correlation network showing the correlation with |R|> 0.30 and p < 0.05 among the gut microbiota, host inflammation and behavioral indicators. **b** Potential gut-brain mechanisms underlying the adverse effect of gut microbiota from ARDS/CAP patients on behavioral functions in mice

To further explore the associations among the gut microbiota, host inflammation and behavioral indicators, correlation network analysis was performed in this study. The correlation with |R|> 0.30 and p < 0.05 was presented in Fig. 6a, where we can see a complex microbiota-gut-brain crosstalk. Our results reveal that behavior changes of mice were closely related to the gut microbiota, colonic barrier functions and host inflammation. For example, the percentage of distance in goal area (DG) in the MWM was positively correlated with cortex ZO-1 and occludin levels but negatively linked to cortex IL-1β and the gut microbes, *Escherichia-Shigella* and *Lactobacillus* (Fig. 6a). A reduction in the percentage of time in goal area (TG) in the MWM in ARDS-R mice was attributed to decreased cortex occludin and *Blautia* as well as increased colonic IL-6 and *Escherichia-Shigella*. Hippocampal TNF-α was negatively related to platform frequency (PF) in the MWM and spontaneous alternation (SA) in the YMT (Fig. 6a). The percentage of distance in center area (DC) in the OFT was positively associated with cortex occludin but negatively with hippocampal IL-6 and colonic ZO-1. Moreover, we found that total distance (TD) in the OFT had a negative correlation with cortex TNF-α and positive correlations with cortex occludin and hippocampal ZO-1, as shown in Fig. 6a.

## Discussion

In this study, we revealed that the gut microbiota from ARDS/CAP patients may result in neuroinflammation and behavioral disorders in mice (Fig. 6b). The gut microbiota was disordered in patients with ARDS/CAP as characterized by enriched abundances of Gram-negative bacteria, causing a higher LPS production. ARDS/CAP microbiota increased the inflow of LPS into the circulation by disrupting colonic barrier integrity of mice and leaded to systemic inflammation. Moreover, blood–brain barrier (BBB) was also disrupted and caused disorders in multiple brain cells of mice including microglia activation, astrocyte proliferation and neuron loss, and eventually impaired behavioral functions (Fig. 6b).

Leaky gut has been regarded as an important pathogenic factor for both intestinal and extra-intestinal diseases, since the damaged intestinal barrier cannot protect against harmful luminal contents into the circulation such as pathogens and microbial toxins [16, 17]. The leaky gut might be attributed to a variety of issues including physical stress, inflammatory disease, dietary factors and pathogenic infections [16]. Herein we revealed that ARDS/CAP patients may also exhibit a leaky gut symptom as indicated by significantly increased circulating levels of two intestinal permeability indicators, DAO and LPS. In fact, the leaky gut has been found as a major pathological characteristic in ARDS patients due to sepsis or COVID-19 [18, 19]. Interestingly, we found that increased colonic permeability can be induced in recipient mice via FMT from ARDS/CAP patients, suggesting a link between gut microbiome and permeability [20]. Of note, the intestinal flora dysbiosis and leaky gut have been proven to promote the progression and severity of many diseases such as COVID-19 [21], kidney disease [22], acute coronary syndrome [23], liver fibrosis [24] and metabolic disease [25]. Besides the leaky gut, the breakdown of BBB, known as “leaky brain”, also occurred in mice receiving the gut microbiota from ARDS/CAP patients as indicated by lower expression levels of tight junction proteins in the cortex and hippocampus. This could be due to the fact that the leakage of the intestinal barrier can increase systematic inflammatory responses and thereby lead to the leaky brain and neurologic dysfunction [26–28]. The leaky gut and brain have been associated with brain diseases such as Alzheimer’s disease [29], Parkinson’s disease [30] and depression [31]. These factors might be also responsible for behavioral disorders in ARDS/CAP patients. Thus, we suggest that the gut microbial modification by some means to enhance the intestinal barrier may be important in understanding the pathogenesis of neuroinflammation and cognitive dysfunction in ARDS patients.

Another finding herein is that both ARDS/CAP patients and mice receiving their gut microbiota had significantly higher Gram-negative bacteria, a primary source of LPS [32]. LPS as a classic pro-inflammatory factor has been reported to induce systemic inflammation and aggravate the progression of diseases including neurodegeneration [33–35]. Once the leaky gut has happened, LPS will pour into the bloodstream and then cause systemic inflammation. In the present study, this undesirable phenomenon was observed in mice receiving ARDS/CAP microbiota including higher circulating levels of LPS and inflammatory cytokines, colonic inflammation as well as lung inflammation and macrophage activation. These results indicate that the gut microbiota dysbiosis played an important role in systemic inflammatory responses in ARDS/CAP patients. In addition, of note, LPS can result in the leaky brain [36, 37], which might be responsible for microglia activation and astrocyte proliferation in the brain of mice receiving ARDS/CAP microbiota. Abnormal astrocyte and microglia further promote neuroinflammation and neuron loss [38, 39], leading to behavioral disorders in ARDS/CAP patients. Therefore, our study proposes that the microbiota-derived LPS acted as an important signaling molecule in the microbiota-gut-brain communication to affect behavioral functions in ARDS/CAP patients.

## Conclusions

Herein we provided evidence that the gut microbiota from ARDS/CAP patients can induce neuroinflammation and behavioral disorders in mice via the gut-brain axis. On the one hand, the enrichment of Gram-negative bacteria produced more LPS and then facilitated inflammatory response. On the other, the leaky gut and brain can accelerate LPS inflow into the circulation, resulting in systemic inflammation, brain disorders and eventually behavioral dysfunctions. In clinical practice, therefore, we suggest that targeting both the intestinal microbiota and leaky gut might be a promising strategy for the prevention and treatment of ARDS/CAP-associated behavioral disorders.

## Materials and methods

### Clinical sample collection

In this study, participants including ARDS patients and healthy subjects were recruited from the First Affiliated Hospital of Wenzhou Medical University. ARDS was diagnosed according to the Berlin definition [40] within 24 h of admission to the hospital and moderate ARDS patients were recruited: (a) acute onset; (b) 100 mm Hg < PaO_2_/FIO_2_ ≤ 200 mm Hg with PEEP ≥ 5 cm H_2_O; (c) bilateral lung opacities on chest radiograph not fully explained by effusion, collapse or nodules; (d) respiratory failure not fully explained by heart failure or fluid overload. Herein ARDS was considered to be caused by CAP, because all patients acquired infection in the community and were hospitalized due to pneumonia. Age-matched healthy volunteers were enrolled without the history of cardiac or respiratory disease and with normal lung functions (FEV1 > 80% predicted; FEV_1_/FVC ratio > 0.7). All participants were excluded from this study if they had received any antibiotics, probiotics and prebiotics more than one week, or had a history of gastrointestinal (GI) diseases including inflammatory bowel disease, GI cancers or GI surgeries. Finally, a total of 21 ARDS/CAP patients and 21 healthy subjects were involved in the current study. Then, faecal samples were collected into sterilized 15 mL EP tubes on the day of admission prior to oral antibiotic treatment and stored at − 80 °C until use. Blood samples were collected into a 5 mL vacutainer tube containing the chelating agent ethylene diamine tetra acetic acid (EDTA), and centrifuged at 1500 g for 15 min to collect serum and kept at − 80 °C until use. This study was reviewed and approved by the Ethics Committee of the First Affiliated Hospital of Wenzhou Medical University, and the written informed consent was obtained from all participants. All procedures in this study are carried out according to the 2008 Helsinki Declaration and the Clinical Ethics Guide of the First Affiliated Hospital of Wenzhou Medical University.

### Animals

Male C57BL/6 mice aged 6 weeks (body weight: 18–22 g) were purchased from the Charles River Laboratory Animal Technology Co., Ltd. (Beijing, China). All mice were housed in a specific pathogen-free (SPF) colony under a fully controlled condition (humidity: 50–60%; temperature: 22 ± 1 °C; light/dark cycle: 12 h/12 h; lights on at 8:00a.m.) at the Laboratory Animal Center of Wenzhou Medical University (Wenzhou, China). Standard mouse chow and tap water were freely available to all animals. All experiments in this study was carried out according to the Guide for the Care and Use of Laboratory Animals and approved by the Institutional Animal Care and Use Committee of Wenzhou Medical University (ID: xmsq2022-0714).

### Fecal microbiota transplantation (FMT)

After one week of acclimation, all mice were randomly divided into two groups where mice were transferred with the faecal microbiota from either ARDS/CAP patients (ARDS-R, n = 13) or normal controls (CTRL-R, n = 13). Prior to FMT, all mice were administered by gavage with an antibiotic cocktail comprising amphotericin B (1 mg/kg), neomycin (100 mg/kg), vancomycin (50 mg/kg) and metronidazole (100 mg/kg) once a day and also fed with drinking water with ampicillin (1 g/L) for 1 week to eliminate the indigenous intestinal microbiota. In this study, fresh faecal samples from volunteers recruited in this study were collected into sterilized 15 mL EP tubes and added with sterilized PBS buffer at a ratio of 1 g/7.5 mL. The mixture was vortexed vigorously for 1 min and then centrifuged at 1000 g for 5 min at 4 °C to prepare fecal bacterial suspension. Then, 100 μL of the bacterial suspension was transplanted to the mice by gavage once a day for 4 weeks.

### Morris water maze test

After 4 weeks of FMT, the Morris water maze (MWM) test was performed to evaluate learning and memory abilities of mice according to our previously reported method [41]. In brief, the MWM test was conducted in a circular pool with a diameter of 110 cm and a height of 30 cm and filled with opaque water by adding nontoxic dye at 26 ± 1 °C. The escape platform (diameter = 7 cm) was submerged 1 cm below the water surface in goal area. During a 4-day training period, mice were guided to reach the escape platform and stay for 15 s by an operator, if they cannot reach the platform within 60 s. At day 5 of the MWM test, the escape platform was removed and the trained mice were subjected to a 90 s space exploration. The swimming trajectory was tracked and recorded by an overhead video camera and a computer system with Viewer-2 software (Biobserve GmbH, Bonn, Germany). The percentages of distance and time in goal area and platform frequency were calculated as indicators of cognitive functions.

### Open field test

In this study, open field test (OFT) was carried out to assess anxiety-like behavior of mice according to a previous method [42]. Briefly, the OFT was conducted in an open-field apparatus with size 40 × 40 × 40 cm and the chamber was divided into 16 small squares with size 10 × 10 cm. Mice were placed in the center of the apparatus and subjected to a 5-min free exploration. The center area was set as a square with 10 cm from the wall. The movement trajectory was recorded by using an overhead video system (DigBehav, Jiliang Co. Ltd., Shanghai, China) and the percentages of distance and time in central area and total distance were calculated in this study.

### Y maze test

Spatial working and memory abilities of mice were examined by using the Y maze test (YMT) [42]. Briefly, the YMT was performed with a Y maze apparatus with size 30 × 50 × 15 cm for each arm. Mice were placed in the center area and subjected to a 5-min free exploration. The movement trajectory was recorded by an overhead video system (DigBehav, Jiliang Co. Ltd., Shanghai, China) and then the percentage of spontaneous alternation and total alternation were calculated.

### Sample collection

Prior to behavioral tests, faecal samples were collected into 1.5 mL sterile EP tubes and kept under − 80℃ until use. Mice with one day rest after behavioral tests were anaesthetized with isoflurane and sacrificed by rapid decapitation. Blood sample was collected and centrifuged at 1500 g at 4 °C for 15 min to separate serum. Subsequently, colon, lung, cortex and hippocampus tissues were promptly collected, frozen in liquid nitrogen and kept at − 80 °C until analysis.

### 16S rRNA sequencing analysis

Total DNA in the fecal microbiota was extracted by using TIANamp stool DNA kit according to the manufacturer’s instruction (TianGen, China). The agarose gel electrophoresis (AGE, 1%) was used to determine the concentration of total DNA. The V3-V4 regions of 16S rRNA gene were amplified with the universal primers 515F (5′-GTG CCA GCM GCC GCG GTA A-3′) and 806R (5′-GGA CTA CHV GGG TWT CTA AT-3′). Subsequently, the PCR product was purified using the QIAquick gel extraction kit on the basis of the manufacturer’s instruction (Qiagen, Germany) and sequenced with an Illumina HiSeq2500 PE250 sequencer (San Diego, USA) at Novogene (Beijing, China).

The raw tags were filtered by merging paired-end reads with FLASH software (v1.2.7) and merged into clean tags by QIIME2 analysis [43]. The effective tags were obtained using UCHIME algorithm (v7.0.1001) and clustered to operational taxonomic units (OTUs) at a similarity cut-off of 97% with UPARSE pipeline (v7.0.1001) [44]. The OTUs were annotated according to taxonomic information in the SILVA database via the Mothur method at a confidence threshold of 80%. Finally, the α- and β-diversity of the gut microbiota were analyzed by QIIME2 software and R software (v3.5.3).

### Histological analysis

In this study, the mice (n = 3) were anaesthetized with isoflurane and sacrificed after normal saline perfusion. The colon, lung and brain tissue samples were collected and fixed with 4% paraformaldehyde in PBS buffer (0.1 M, pH = 7.5). The tissue samples were dehydrated with a graded series of ethanol, embedded in paraffin and sectioned into 5 μm slices using a slicing machine (Leica, Germany). To observe the changes in brain cells, the brain sections were incubated with Anti-Iba1 antibody (Abcam, ab178846, 1:200) for microglia, Anti-GFAP antibody (CST, 3670 s, 1:200) for astrocyte and Anti-NeuN antibody (CST, 24307 s, 1:400) for neuron overnight at 4 °C. To assess the intestinal barrier integrity, the colonic sections were incubated with primary antibody ZO-1 (ABclonal, 21773-1-AP, 1:50) or Occludin (ABclonal, 27260-1-AP, 1:50) at 4 °C overnight. After washing with PBS buffer, the sections were incubated with Alexa FlourTM 488 goat anti-rabbit lgG (H + L; Invitrogen, 1:400) at 37 °C for 1 h, and then washed with PBS buffer and added with DAPI (Southern Biotech, AL, USA) for 2 min at 22 °C. The images were captured by using a confocal microscope (A1R-SIMSTORM, Nikon, Japan) and the quantitative analysis was performed by Image J software (v 1.47, Bethesda, MD). To obtain the histopathological changes, the colonic and lung sections were dewaxed, rehydrated with gradient alcohol, stained in hematoxylin for 3 min and washed with tap water. Then, the sections were differentiated in 95% ethyl alcohol for 5 min, stained in 1% Eosin for 10 min, washed with tap water and then mounted in neutral resin. To observe lung macrophages, the lung sections were dewaxed in xylene, rehydrated with gradient alcohol, and stained with F4/80 antibody. After washing, the sections were differentiated in 95% ethyl alcohol for 5 min, dehydrated in alcohol, cleared in xylene and then mounted with neutral resin. Finally, the images were captured at 200 × magnification with an optical microscope (Nikon, Japan) and the quantitative analysis was carried out by Image J software.

### Quantitative real-time PCR analysis

Total RNA in the tissue samples was isolated using Trizol reagent (Invitrogen, USA) according to the manufacturer’s protocol. The purity of total RNA was measured on a NanoDrop spectrometer (Thermo Fisher Scientific, Beverly, MA). Then cDNA samples were synthesized by HiScript III RT SuperMix for qPCR (+ gDNA wiper) (Vazyme) in line with the manufacturer’s instruction. The quantification analysis was performed using SYBR Premix Ex Taq II kit (Takara, Dalian, China) on Bio-Rad CFX384. The specific primer pairs were synthesized from Tsingke Biotechnology (Beijing, China) as listed in Additional file 1: Table S3. The relative mRNA expression level was normalized to GAPDH and calculated using the ΔΔCT method.

### Analysis of serum LPS, diamine oxidase and inflammatory cytokines by ELISA

The levels of lipopolysaccharide (LPS), diamine oxidase (DAO) and inflammatory cytokines including IL-2, IL-4, IL-6 and TNF-α were measured by ELISA kits from Nanjing Jiancheng Bioengineering Institute (Nanjing, China) according to the manufacturer’s instructions.

### Statistical analysis

In this study, all mice were randomly assigned to experiments including animal grouping, FMT operation, sample collection and data analysis. The difference of the gut microbial pattern between two groups was analyzed by using principal coordinate analysis (PCoA) based on Bray–Curtis distance in R software (v2.15.3). The statistic difference of various indicators between two groups was evaluated by two-tailed unpaired student’s T test in SPSS 22.0 software (SPSS, Inc., Chicago, IL, USA). The statistic difference in the change of the escape latency between two groups was analyzed via a repeated measure ANOVA in SPSS software. Herein a statistically significant was defined when p < 0.05. A volcano plot was performed by plotting log_2_ (fold change) versus –log_10_ (p value) with R software (v2.15.3). The relationship between different indicators was analyzed by Spearman’s correlation and correlation network was visualized with the Cytoscape software (v3.9.1) [45].

## Supplementary Information

**Additional file 1: Table S1.** Primer pairs used in this study. **Table S2.** Clinical characteristics of participants. **Table S3.** The number of significantly altered gut microbes between ARDS/CAP patients and normal controls. **Figure S1.** The gut microbiota from ARDS/CAP patients leads to lung inflammation in mice. **Figure S2.** The gut microbiota from ARDS/CAP patients causes astrocyte proliferation and neuron loss in the brain of mice.

**Additional file 2:** The significantly altered gut microbes between ARDS/CAP and CTRL subjects.

## Data Availability

All data used in this study are present in the main text and additional files. Additional data and materials can also be requested from corresponding author.

## Abbreviations

ARDS: Acute respiratory distress syndrome
CAP: Community-acquired pneumonia
CTRL: Healthy controls
FMT: Faecal microbiota transplantation
LPS: Lipopolysaccharide
MWM: Morris water maze
OFT: Open field test
YMT: Y-maze test
