# Supplementary material for: Gastrointestinal microbiome of ARDS patients induces neuroinflammation and cognitive impairment in mice
Source: J Neuroinflammation. 2023 Jul 15;20:166. doi: 10.1186/s12974-023-02825-7 (PMC10349492; doi:10.1186/s12974-023-02825-7)
Supplement: Supplementary file 1 — Additional file 1: Table S1. Primer pairs used in this study. Table S2. Clinical characteristics of participants. Table S3. The number of significantly altered gut microbes between ARDS/CAP patients and normal controls. Figure S1. The gut microbiota from ARDS/CAP patients leads to lung inflammation in mice. Figure S2. The gut microbiota from ARDS/CAP patients causes astrocyte proliferation and neuron loss in the brain of mice. [file 12974_2023_2825_MOESM1_ESM.docx]

Gastrointestinal microbiome of ARDS patients induces neuroinflammation and cognitive impairment in mice

Running Title: ARDS/CAP microbiota causes brain disorders in mice

Hong Zheng^1,†^, Qihui Zhao^1,†^, Jianuo Chen^2^, Jiahui Lu^1^, Yuping Li^2,*^ & Hongchang Gao^1,*^

^1^Oujiang Laboratory, School of Pharmaceutical Sciences, Wenzhou Medical University, Wenzhou 325035, China

^2^Department of Pulmonary and Critical Care Medicine, The First Affiliated Hospital of Wenzhou Medical University, Wenzhou 325015, China

*Corresponding author: [wzliyp@163.com](mailto:wzliyp@163.com) (Y.P.L.); [gaohc27@wmu.edu.cn](mailto:gaohc27@wmu.edu.cn) (H.C.G.).

^†^These authors contributed equally.

**Table S1.** Clinical characteristics of participants.

| Item | ARDS/CAP^a^ (n=21) | CTRL^b^ (n=21) | Normal range |
| --- | --- | --- | --- |
| Sex (M/F) | 15/6 | 14/7 | p=0.7 |
| Age (year) | 66.0±3.4 | 63.9±3.3 | p=0.6 |
| Procalcitonin (ng/mL) | 17.7±4.6 | - | 0.0-0.5 |
| C-reactive protein (mg/L) | 221.2±10.8 | - | 0.0-6.0 |
| IL-2 (μg/L) | 0.7±0.8 | - | 0.0-3.0 |
| IL-4 (μg/L) | 0.3±0.6 | - | 0.0-3.0 |
| IL-6 (μg/L) | 6454.3±107.6 | - | 0.0-3.0 |
| TNF-α (pg/mL) | 1.4±1.3 | - | 0.0-3.0 |
| DAO^c^ (ng/mL) | 77.3±15.7 | 52.3±5.5 | p=0.001 |
| LPS^d^ (pg/mL) | 267.1±30.9 | 202.2±58.7 | p=0.015 |

^a^ Acute respiratory distress syndrome caused by community-acquired pneumonia; ^b^ Normal control subjects; ^c^ Diamine oxidase; ^d^ Lipopolysaccharide.

**Table S2.** The number of significantly altered gut microbes between ARDS/CAP patients and normal controls.

|  | UP^a^ | DOWN^b^ | Total |
| --- | --- | --- | --- |
| Firmicutes | 22 (44.90%) | 27 (55.10%) | 49 |
| Proteobacteria | 29 (93.55%) | 2 (6.45%) | 31 |
| Bacteroidota | 10 (83.33%) | 2 (16.67%) | 12 |
| Actinobacteriota | 17 (100.00%) | 0 (0.00%) | 17 |
| Others | 33 (94.29%) | 2 (5.71%) | 35 |
| Total | 111 (77.08%) | 33 (22.92%) | 144 |

^a^ Significantly higher in ARDS/CAP patients relative to normal controls;

^b^ Significantly lower in ARDS/CAP patients relative to normal controls.

**Table S3.** Primer pairs used in this study.

| Gene | Forward primer | Reverse primer |
| --- | --- | --- |
| IL-6 | CAGCATGGCTGATGTTAAGTTTTC | CCCAGGCAGGCGCATAC |
| TNF-α | GACGTGGAACTGGCAGAAGAG | TTGGTGGTTTGTGAGTGTGAG |
| IL-1β | GCAACTGTTCCTGAACTCAACT | ATCTTTTGGGGTCCGTCAACT |
| occludin | TTGAAAGTCCACCTCCTTACAGA | CCGGATAAAAAGAGTACGCTGG |
| ZO-1 | GCCGCTAAGAGCACAGCAA | TCCCCACTCTGAAAATGAGGA |
| GAPDH | AGGTCGGTGTGAACGGATTTG | TGTAGACCATGTAGTTGAGGTCA |


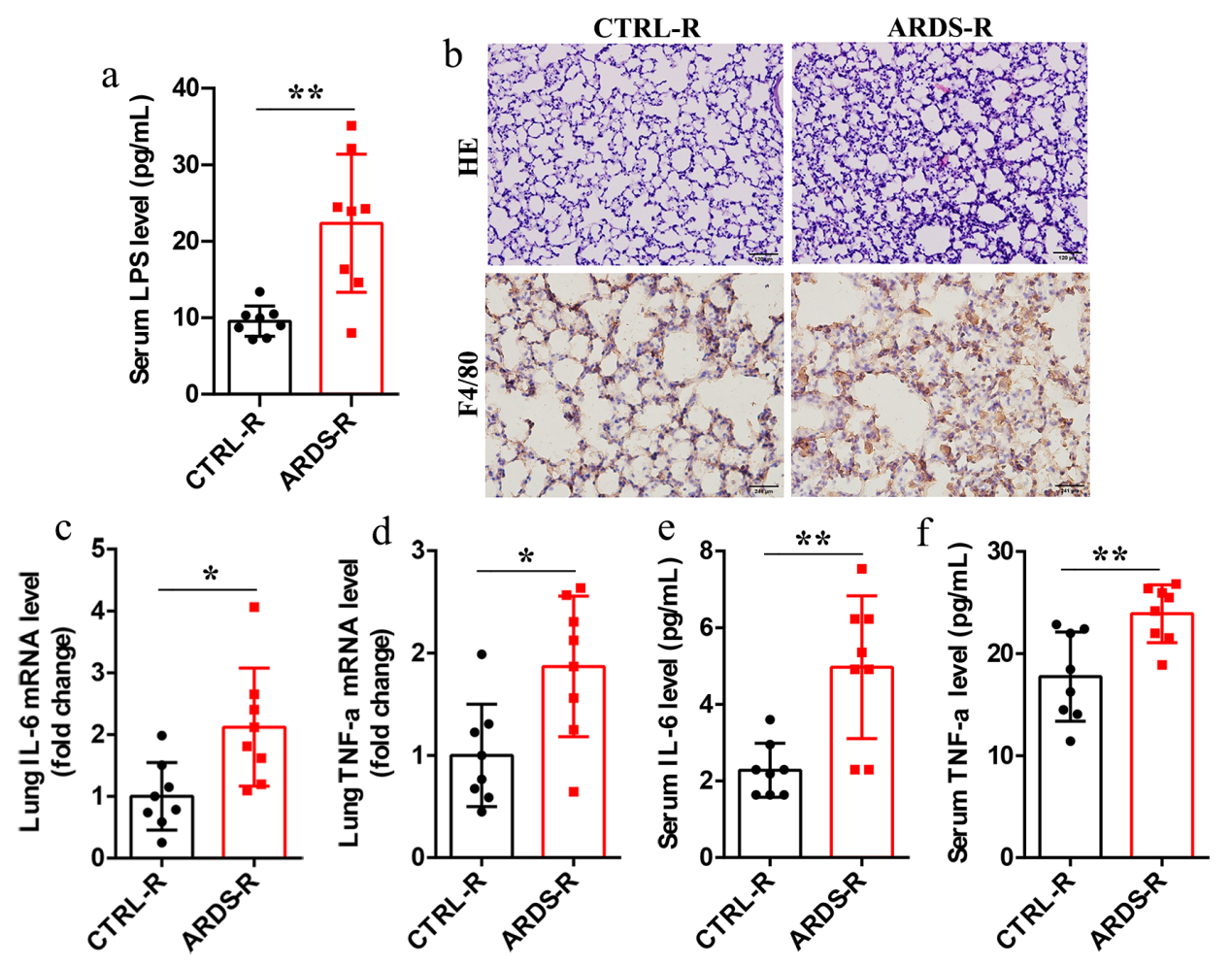


**Figure S1.** The gut microbiota from ARDS/CAP patients leads to lung inflammation in mice. (a) Changes in serum lipopolysaccharide (LPS) level in mice receiving the faecal microbiota from ARDS/CAP patients (ARDS-R) or normal control subjects (CTRL-R). (b) Hematoxylin/Eosin (HE) staining and F4/80 immunostaining showing the histological change and M1 macrophage activation in the lung of ARDS-R and CTRL-R mice. (c, d) The mRNA expression levels of lung (c) IL-6 and (d) TNF-α. (e ,f) The mRNA expression levels of serum (e) IL-6 and (f) TNF-α. The statistic difference of various indicators between two groups was evaluated by two-tailed unpaired student’s T test and a statistically significant was defined when p<0.05. Significant level: *p<0.05; **p<0.01.


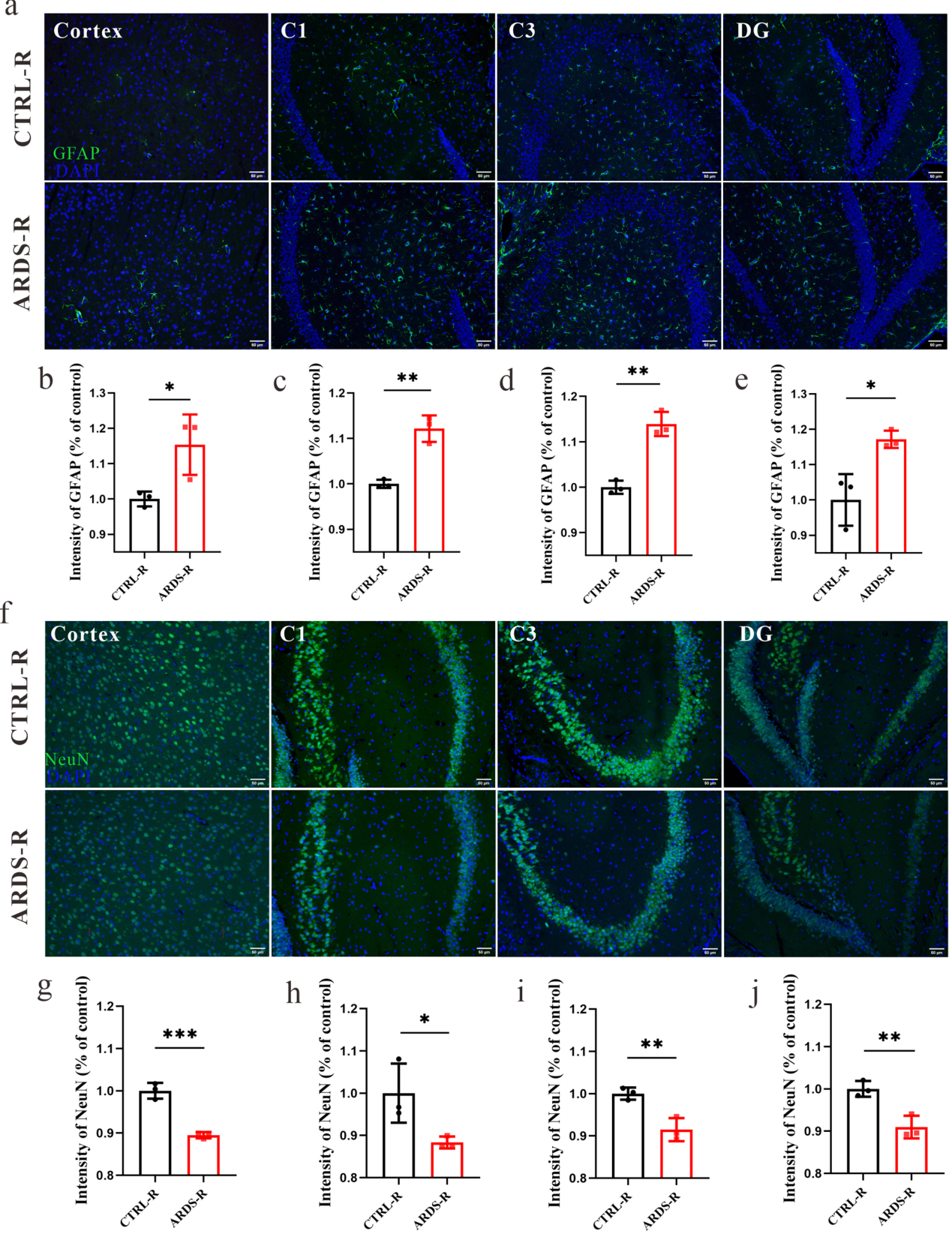


**Figure S2.** The gut microbiota from ARDS/CAP patients causes astrocyte proliferation and neuron loss in the brain of mice. (a) Typical GFAP immunostaining showing the number of astrocytes in the cortex and different hippocampal regions including C1, C3 and DG of mice receiving the faecal microbiota from ARDS/CAP patients (ARDS-R) or normal control subjects (CTRL-R). (b-e) The corresponding quantitative results of GFAP positive cells in the (b) cortex and hippocampal (c) C1, (d) C3 and (e) DG regions of ARDS-R and CTRL-R mice. (f) Typical NeuN immunostaining showing the number of neurons in the cortex and hippocampal C1, C3 and DG regions of ARDS-R and CTRL-R mice. (g-j) The corresponding quantitative results of NeuN positive cells in the (g) cortex and hippocampal (h) C1, (i) C3 and (j) DG regions of ARDS-R and CTRL-R mice. The statistic difference of various indicators between two groups was evaluated by two-tailed unpaired student’s T test and a statistically significant was defined when p<0.05. Significant level: *p<0.05; **p<0.01; ***p<0.001.
